# Análise do Teste de Esforço Cardiopulmonar no Pré e Pós-COVID-19

**DOI:** 10.36660/abc.20250544

**Published:** 2025-10-28

**Authors:** José Antônio Caldas Teixeira

**Affiliations:** 1 Universidade Federal Fluminense Rio de Janeiro RJ Brasil Universidade Federal Fluminense, Rio de Janeiro, RJ – Brasil; 2 Clínica Fit Center Rio de Janeiro RJ Brasil Clínica Fit Center, Rio de Janeiro, RJ – Brasil

**Keywords:** Teste de Esforço, Aptidão Física, Síndrome de Pós-COVID-19 Aguda

A pandemia de SARS-CoV-2 (COVID-19), que começou em dezembro de 2019 na cidade de Wuhan, província de Hubei, China, rapidamente se tornou uma pandemia, afetando mais de 600 milhões de pessoas, resultando em mais de 6 milhões de mortes. A COVID-19 tem sido associada a mortalidade e morbidade significativas, incluindo sequelas pulmonares e cardíacas adversas.^1^

Sabe-se que os sintomas da COVID-19 persistem além da fase aguda, levando a sequelas pós-agudas, conhecidas como Síndrome da COVID Longa, que duram mais de 60 dias ou indefinidamente.^2^ Embora os mecanismos precisos não estejam totalmente esclarecidos, ela está associada a vários sintomas, sendo a fadiga e a intolerância ao exercício os mais prevalentes.^2^

O teste cardiopulmonar de exercício (TCPE) é a ferramenta padrão-ouro para identificar as causas da intolerância ao exercício.^3^ Estudos que utilizaram o TCPE para avaliar o período pós-COVID-19 detalharam diversas alterações, como redução da capacidade funcional, anaerobiose precoce e diversas alterações na ventilação pulmonar. No entanto, esses estudos, embora limitados em número, geralmente incluíram pacientes hospitalizados por formas moderadas a graves da doença. Identificaram redução da capacidade funcional relacionada principalmente a fatores periféricos (extração de oxigênio) e nem sempre a uma limitação respiratória ou cardíaca, desde que não houvesse sequelas evidentes desses sistemas (p. ex., fibrose pulmonar, disfunção pós-miocardite).^4,5^

No pico da pandemia, diversas diretrizes foram emitidas para avaliar o retorno dos atletas à prática esportiva, incluindo o TCPE nesse fluxo, inclusive com produções nacionais.^6,7^ A prática regular de atividade física e/ou exercícios, com consequente melhora do condicionamento físico, têm sido citados como protetores contra as formas mais graves da COVID-19.^8^

No entanto, nesta avaliação pré e pós-COVID-19, até o momento, houve apenas um número limitado de estudos que realizaram um TCPE pré e pós-COVID-19 em indivíduos mais ativos. O estudo de Braga et al.^9^ ajuda a preencher essa lacuna.

O estudo^9^ teve como objetivo comparar os achados metabólicos e ventilatórios de um TCPE realizado antes e depois da COVID-19 em indivíduos altamente ativos (IAA). Este foi um estudo transversal com análise ex post facto dos dados do TCPE em indivíduos maiores de 18 anos, considerados altamente ativos fisicamente pela escala de Saltin-Grimby, que realizaram TCPE anteriormente e foram reavaliados após a COVID-19 antes de retomar o treinamento.^9^

Os autores reconhecem algumas limitações, como a inclusão apenas de casos leves de COVID-19, a ausência de um grupo controle, a combinação do uso de diferentes ergômetros (cicloergômetro vs. esteira), a ausência de análises específicas por sexo e o envolvimento de IAA, mas não de atletas de elite. No entanto, eles descrevem resultados que levantam pontos de análise interessantes a serem considerados.

Os autores observaram que não houve mudanças significativas nas variáveis espirométricas, mas apareceram alterações em alguns parâmetros do TCPE. O consumo de oxigênio (VO2) no Limiar Ventilatório 2 (LV1) e o consumo de oxigênio de pico (VO2 de pico) foram reduzidos. Essa redução no VO2 de pico pós-COVID-19 ficou abaixo do limite de −13% para significância clínica; no entanto, 14% da amostra apresentou redução significativa nessa variável.

Em relação às variáveis relacionadas à eficiência ventilatória, apenas a razão de pico entre ventilação por minuto e produção de dióxido de carbono (VE/VCO2) aumentou significativamente, sem outras diferenças observadas. O pico delta da relação VE/VCO2 e da frequência respiratória no Limiar Ventilatório 2 (LV2) diminuiu ligeiramente após a COVID-19, mas essas alterações não tiveram significado clínico ou biológico. Não puderam ser claramente atribuídas aos efeitos pós-COVID-19 ou a uma redução na carga de treinamento durante a recuperação.

O estudo^9^ revelou pequenas alterações no pico de VO2 e LV2, e um ligeiro aumento na relação pico de VE/VCO2. Embora estatisticamente significativas, essas alterações não foram conclusivas em relação a distúrbios cardiopulmonares graves, pois estavam abaixo dos limites de diferença crítica, sugerindo que podem não ser diretamente atribuíveis a alterações cardiopulmonares causadas pela COVID-19.

Outros autores relataram resultados semelhantes, como Śliż et al.,^10^ Parpa et al.,^11^ mas os dados contradizem D’Isabel et al.,^12^ que encontraram alterações significativas tanto no pico de VO2 quanto no VO2 no VT1 após COVID-19.

O estudo^9^ fornece insights adicionais com uma proporção maior de participantes do sexo feminino (27,9% vs. 12,2%) e um intervalo menor entre COVID-19 e TCPE, enriquecendo a compreensão do impacto da COVID-19 na função cardiopulmonar.

O estudo,^9^ acrescenta outra fonte de análise usando TCPE antes e depois da COVID-19, demonstrando a resiliência do corpo humano a esta doença, particularmente em uma população altamente ativa, destacando o potencial efeito protetor de um estilo de vida ativo contra as graves repercussões da COVID-19, que, apesar das cepas menos agressivas, continua a circular em nosso meio ambiente.
