# Supplementary material for: Análise do Teste de Esforço Cardiopulmonar no Pré e Pós-COVID-19
Source: Arq Bras Cardiol. 2025 Oct 28;122(9):e20250544. [Article in Portuguese] doi: 10.36660/abc.20250544 (PMC12671596; doi:10.36660/abc.20250544)
Supplement: Translation

# Translation: Analysis of Cardiopulmonary Exercise Test in Pre- and Post-COVID-19

**DOI:** 10.36660/abc.20250544i

**Authors:** José Antônio Caldas Teixeira

**Affiliations:** 1 Rio de Janeiro RJ Brazil Universidade Federal Fluminense, Rio de Janeiro, RJ – Brazil; 2 Rio de Janeiro RJ Brazil Clínica Fit Center, Rio de Janeiro, RJ – Brazil

**Keywords:** Exercise Test, Physical Fitness, Post-Acute COVID-19 Syndrome

The SARS-CoV-2 (COVID-19) pandemic that began in December 2019 in the city of Wuhan, Hubei Province, China, quickly became a pandemic affecting over 600 million people, resulting in over 6 million deaths. COVID-19 has been associated with significant mortality and morbidity, including adverse pulmonary and cardiac sequelae.^1^

It is known that COVID-19 symptoms persist beyond the acute phase, leading to post-acute sequelae, known as Long COVID Syndrome, which lasts for more than 60 days or indefinitely.^2^ Although the precise mechanisms are not fully elucidated, it is associated with various symptoms, with fatigue and exercise intolerance being the most prevalent.^2^

The cardiopulmonary exercise test (CPET) is the gold standard tool for identifying the causes of exercise intolerance.^3^ Studies that have used CPET to evaluate post-COVID-19 have detailed various changes, such as reduced functional capacity, early anaerobiosis, and various pulmonary ventilation alterations. However, these studies, although limited in number, were generally in patients who were hospitalized for moderate to severe forms of the disease. They identified reduced functional capacity mainly related to peripheral factors (oxygen extraction) and not always to a respiratory or cardiac limitation, provided there were no clear sequelae from these systems (e.g., pulmonary fibrosis, post-myocarditis dysfunction).^4,5^

At the peak of the pandemic, various guidelines were issued for evaluating athletes returning to sports practice, including CPET in this flow, including national productions.^6,7^ Regular physical activity and/or exercise, with consequent improved physical fitness, have been cited as protection against the more severe forms of COVID-19.^8^

However, in this pre- and post-COVID-19 evaluation, to date, there have been only a limited number of studies that have conducted a CPET pre- and post-COVID-19 in more active individuals. The study by Braga et al.^9^ helps to fill this gap.

The study^9^ aimed to compare the metabolic and ventilatory findings of a CPET performed before and after COVID-19 in highly active individuals (HAI). This was a cross-sectional study with an ex post facto analysis of CPET data in individuals over 18 years old, considered highly physically active according to the Saltin-Grimby scale, who had a previous CPET and were reassessed post-COVID-19 before resuming training.^9^

The authors acknowledge some limitations, such as including only mild cases of COVID-19, the absence of a control group, mixing the use of different ergometers (cycle ergometer vs. treadmill), the absence of specific sex analyses, and involving HAI, but not elite athletes. However, they describe findings that raise interesting analysis points to be considered.

The authors observed that there were no significant changes in the spirometry variables, but alterations appeared in some CPET parameters. The oxygen consumption (VO2) at Ventilatory Threshold 2 (VT1) and peak oxygen consumption (Peak VO2) were reduced. This reduction in peak VO2 post-COVID-19 was below the −13% limit for clinical significance; however, 14% of the sample had a significant reduction in this variable.

Regarding variables related to ventilatory efficiency, only the peak ratio of minute ventilation to carbon dioxide production (VE/VCO2) significantly increased, without any other differences noted. The delta peak of the VE/VCO2 ratio and respiratory rate at Ventilatory Threshold 2 (VT2) decreased slightly after COVID-19, but these changes lacked clinical or biological significance. They could not be clearly attributed to post-COVID-19 effects or to a decrease in training load during recovery.

The study^9^ revealed small changes in peak VO2 and VT2, and a slight increase in the peak VE/VCO2 ratio. Although statistically significant, these changes were not conclusive regarding major cardiopulmonary disturbances, as they were below critical difference thresholds, suggesting that they may not be directly attributable to cardiopulmonary changes caused by COVID-19.

Other authors have reported similar findings, such as Śliż et al.,^10^ Csulak et al.,^11^ but the data contradicted D’Isabel et al.,^12^ who found significant alterations in both Peak VO2 and VO2 at VT1 after COVID-19.

The study^9^ provides additional insights with a higher proportion of female participants (27.9% vs. 12.2%) and a shorter interval between COVID-19 and CPET, enriching the understanding of the impact of COVID-19 on cardiopulmonary function.

The study,^9^ adds another source of analysis using CPET before and after COVID-19, demonstrating the resilience of the human body to this disease, particularly in a highly active population, highlighting the potential protective effect of an active lifestyle against the severe repercussions of COVID-19, which, despite the less aggressive strains, continues to circulate in our environment.
